# Eating Away at Antibiotic Resistance – Is Phage Therapy Our Future?

**DOI:** 10.20411/pai.v11i1.967

**Published:** 2026-02-09

**Authors:** Michael M. Lederman, Robert A. Bonomo

**Affiliations:** 1 Case Western Reserve University School of Medicine, Cleveland, Ohio; 2 Medical Service, Louis Stokes Cleveland VA, and Case Western Reserve University, Cleveland, Ohio

**Keywords:** Bacteriophages, Phage Therapy, Antibiotic Resistance, Bacterial Infections, Multidrug-resistant Bacteria

In 1917, French microbiologist Felix d’Herelle recognized that an antibacterial activity first noted by Frederick W. Twort in 1915 was attributable to filtrable agents that infected bacteria; he named these agents “bacteriophages” – “bacteria eaters.” These unusual viruses specifically target and destroy bacteria, offering a natural mechanism for controlling bacterial populations.

Realizing the importance of this, d’Herelle and many others worked to apply bacteriophages for the treatment of serious bacterial infections like typhoid, plague, and cholera. These early therapeutic efforts had inconsistent results as methods for bacteriophage preparation were not standardized, and understanding of phage biology was rudimentary. Interest in the therapeutic application of bacteriophages nearly disappeared as the antibiotic era was introduced in the 1940s. This is rapidly changing as we are faced with the emerging global crisis of antibiotic resistance.

Now, more than 100 years after their discovery, bacteriophages and their therapeutic applications were highlighted in the 2025 Conference on Bacteriophages: Biology, Dynamics, and Therapeutics, which was held this past October in Washington, D.C., and which is summarized in this issue of *Pathogens and Immunity* by Elshazly et al [1]. As more and more serious infections are being caused by bacteria intrinsically resistant or evolved to become resistant to available antibiotics, the study of bacteriophages to treat these infections is increasingly important. Why does this matter? Bacteriophages, unlike antibiotics, selectively target specific bacteria and can also penetrate biofilms. One can easily envision an approach where bacteriophages can complement or even replace antibiotics with little impact on normal flora.

Outlined in this summary are fascinating studies of bacteriophage biology, the determinants and predictors of bacterial susceptibility and resistance to bacteriophages. Most exciting to read are the reports of the use of bacteriophages against multidrug-resistant infections and the basic science that is evolving in this sphere. Central to the broader application of these therapies is the development of standardized methods for testing phage antibacterial activity. Clinical reports and trials in the compromised host and the host with hard-to-treat prosthetic joint infections are also presented, as are studies of therapeutic phage pharmacokinetics and pharmacodynamics.

Readers of *Pathogens and Immunity* will find this summary a welcome update to the 2022 *Pathogens and Immunity* review of bacteriophage therapy by Zagaliotis et al [2]. As these exciting therapies are becoming more practicable for the treatment of drug-resistant infections, bacteriophages look to be in our future. There will be challenges (standardization, regulation, and resistance), but meeting them will be worth the effort.
